# Tumor burden as possible biomarker of outcome in advanced NSCLC patients treated with immunotherapy: a single center, retrospective, real-world analysis

**DOI:** 10.37349/etat.2021.00043

**Published:** 2021-06-28

**Authors:** Edoardo Lenci, Giulia Marcantognini, Valeria Cognigni, Alessio Lupi, Silvia Rinaldi, Luca Cantini, Ilaria Fiordoliva, Anna Lisa Carloni, Marco Rocchi, Lina Zuccatosta, Stefano Gasparini, Rossana Berardi

**Affiliations:** 1 Clinical Oncology, Università Politecnica delle Marche, Azienda Ospedaliero-Universitaria Ospedali Riuniti, 60126 Ancona, Italy; 2 Department of Biomolecular Sciences, University of Urbino Carlo Bo, 61029 Urbino, Italy; 3 Operative Unit of Pneumology, Ospedali Riuniti University Hospital, 60126 Ancona, Italy; Université Côte d’Azur; Nice University Hospital, France

**Keywords:** Immunotherapy, non-small cell lung cancer, tumor burden

## Abstract

**Aim:**

The role of tumor burden (TB) for patients with non-small cell lung cancer (NSCLC) receiving immunotherapy is still unknown. The aim of this analysis was to analyze the prognostic value of TB in a real-world sample of advanced NSCLC patients.

**Methods:**

Sixty-five consecutive patients with advanced NSCLC treated with immunotherapy as first or second line therapy were retrospectively analyzed between August 2015 and February 2018. TB was recorded at baseline considering sites and number of metastases, thoracic *vs*. extrathoracic disease, measurable disease (MD) *vs*. not-MD (NMD) and evaluating dimensional aspects as maximum lesion diameter (cut-off = 6.3 cm), sum of the 5 major lesions diameters (cut-off = 14.3 cm), and number of sites of metastases (cut-off > 4). All cut-offs were calculated by receiver operating characteristic curves. Median overall survival (OS) was estimated using Kaplan-Meier method. A Cox regression model was carried out for univariate and multivariate analyses.

**Results:**

Median age was 70 years and most patients (86.2%) had a good performance status (PS-Eastern Cooperative Oncology Group < 2). No significant difference in OS was noted between subgroups of patients according to TB. Bone metastases (BM) had a negative prognostic impact [median OS (mOS), 13.8 *vs*. 70.0 months, *P* = 0.0009; median progression free survival in the second line (mPFS2) 2.97 *vs*. 8.63 months; *P* = 0.0037]. Patients with NMD had a poorer prognosis (mOS, 15.9 months *vs*. not reached, *P* < 0.0001; mPFS2 3.8 *vs*. 12.2 months; *P* = 0.0199). Patients with disease limited to the thorax had a better prognosis compared to patients with involvement of extrathoracic sites (mOS, 70 *vs*. 17.3 months; *P* = 0.0136). Having more than 4 metastatic sites resulted as a negative prognostic factor (mOS, 15.9 *vs*. 25.2 months; *P* = 0.0106). At multivariate analysis, BM, NMD, extrathoracic disease and number of sites of metastases > 4 were negative prognostic factors (*P* < 0.0001).

**Conclusions:**

This study underlines the negative prognostic impact of specific metastatic sites, presence of NMD and extrathoracic disease in advanced NSCLC patients treated with immunotherapy. However, TB does not appear to affect the outcome of these patients.

## Introduction

Lung cancer is the second most commonly diagnosed cancer in the world, recording 2,206 million new cases, corresponding to 11.4% of new diagnoses [1]. Despite the important results obtained from cancer research, it remains the leading cause of cancer-related death both in men and in women [2].

In the last few years, the advent of immune checkpoint inhibitors (ICIs) has dramatically changed the treatment landscape of advanced non-small cell lung cancer (NSCLC) patients, with a significant impact on both quality of life and overall survival (OS) [3–7]. Nowadays, standard systemic treatment for advanced non oncogene-addicted NSCLC is represented by the combination of chemotherapy and ICIs, which shown to be effective in all subgroups of patients regardless of programmed death-ligand 1 (PD-L1) expression, with the larger benefit being revealed when PD-L1 expression is upper than 50% [7, 8]. According to current approval in Italy, ICIs in combination with chemotherapy are used as first-line treatment for advanced NSCLC patients with PD-L1 expression lower than 50%, while monotherapy with pembrolizumab [a fully humanized immunoglobulin G4 (IgG4) monoclonal antibody against programmed cell death-1 (PD-1)] is indicated in the same setting when PD-L1 expression is ≥ 50% [9].

Despite the success of ICIs, due to tumor heterogeneity, still not all patients derive benefit from this approach and new biomarkers are needed to improve patient selection.

Currently, PD-L1 expression is the only prospectively validated biomarker of immunotherapy response in patients with non-oncogene addicted NSCLC [10]. PS and specific hematologic values, such as neutrophilto-lymphocyte ratio, lactate dehydrogenase (LDH), and serum albumin have been recognized as promising prognostic factors both in patients treated with chemotherapy and with immunotherapy [11–15]. Furthermore, recent research has shown promise for alternative markers of response, including tumor-infiltrating immune cell [16, 17], expression of indoleamine 2, 3-dioxygenase (IDO), microbiome profile, mutational load, mismatch repair deficiency (dMMR), expression of inflammatory genes [18] baseline metabolic tumor volume [19], body mass index [20], smoking status [21] and concomitant medications [22].

The role played by the metastatic site in prognosis prediction of NSCLC patients still represents a matter of debate. In particular, liver metastases seem to be related to a worse outcome, but the role of other metastatic sites such as bone, central nervous system (CNS), lymph nodes, and lung remains controversial [23–25].

Tumor burden (TB) is assessed as the tumor load detected with radiological exams. Albeit the definition of TB is not consistent across studies, it has been hypothesized that a high TB at diagnosis or at the start of first-line treatment may be correlated with a worse prognosis [26–28]. The prognostic value of TB has been investigated in different solid tumors, such as melanoma, colon-rectal cancer, renal cell cancer and hepatocarcinoma, yet its assessment differed in each study [26–28]. In some studies, TB was calculated as the sum of diameters of target lesions, in other cases as the number and diameter of metastases.

The aim of this study is to investigate the potential prognostic value of specific sites of metastasis in a real-world cohort of advanced NSCLC patients, treated with immunotherapy. Thus, we evaluated if TB should play a role as predictor of clinical outcome.

## Materials and methods

### Patient characteristics

In our study, we retrospectively analyzed data from 65 patients with NSCLC treated with first/second-line immunotherapy, at our institution (Clinical Oncology, Azienda Ospedaliero-Universitaria Ospedali Riuniti, Ancona), between August 2015 and February 2018.

#### Inclusion criteria included:

(1) Age > 18 years old;

(2) Eastern Cooperative Oncology Group (ECOG)-PS ≤ 3;

(3) Histologically confirmed diagnosis of NSCLC;

(4) Patients treated with first/second-line immunotherapy with nivolumab, atezolizumab or pembrolizumab;

(5) epithelial growth factor receptor (*EGFR*), ROS proto-oncogene 1 (*ROS-1*) and anaplastic lymphoma kinase (*ALK*) wild type tumor.

#### Exclusion criteria included:

(1) Patients undergoing exclusively chemotherapy and/or third or subsequent lines of immunotherapy;

(2) Pre-existing diseases or condition that contraindicated immunotherapy:

active systemic autoimmune disease;
history of severe hypersensitivity to another monoclonal antibody;
history of severe immune-related adverse reactions from treatment with ICIs or anti-CTLA4;
severe active infections;
conditions of severe immunodeficiency;
use of systemic corticosteroids (equivalent to >10 mg of prednisone/day);

(3) *EGFR*, *ROS-1* or *ALK* mutated tumor.

Depending on the temporal context in which this study has been conducted, our cohort included NSCLC patients who received monotherapy with ICIs as first or second line, before the results of clinical trial on chemo-immunotherapy combination were released. A flow chart of the study population is available in the supplementary material (Figure S1).

### Clinical variables

Collected data included demographic characteristics (gender, age), smoking habit, comorbidities and concomitant therapies, type of surgical intervention (if performed), number and sites of lymph nodes in case of lymphadenectomy, type of adjuvant therapy (if administered), tumor histotype and grading, PD-L1 expression, presence of mutation or rearrangement of *EGFR*, *ALK*, *ROS-1* genes, tumor stage, sites and number of metastases, PS-ECOG at diagnosis and at every different therapy line start, type of chemotherapy or immunotherapy, type of maintenance therapy (if given), number and type of grade 3–4 toxicities, type and site of radiotherapy (if effected), laboratory parameters including hemoglobin, absolute count of neutrophils, lymphocytes, monocytes and eosinophils, platelets, sodium, albumin, LDH and alkaline phosphatase (AP). For all previous values, the cut-offs commonly used in clinical practice were considered.

Tumor response to immunotherapy was assessed every three months from start of treatment and until progression, regardless of the type of ICI (pembrolizumab, atezolizumab, nivolumab) used. All patients underwent a computed tomography (CT)-scan before the start of treatment and then every three months until clinical or radiological progression or death. Tumor response to immunotherapy was assessed by an expert radiologist using IgG4 immune Response Evaluation Criteria in Solid Tumors (iRECIST) criteria and according to approved guidelines [29]. Fluorodeoxyglucose (FDG)-positron emission tomography (PET)/CT evaluation has been performed only in case of lesions suspected for metastases at CT-scan.

### TB

As a universally accepted method still must be found, we decided, according to literature, to determine TB considering:

1. the number and the size of metastases (according to work by Sasaki, et al. [28], TB was defined as the number and size of metastases);
2. the diameters of the main target lesion (according to work by Iacovelli, et al. [26], TB was defined as the major diameters of the target lesions);
3. the total diameters of the 5 biggest target lesions (according to work by Katsurada, et al. [30], TB defined as the sum of the major diameters of the target lesions).

We have evaluated different definitions of TB in a cohort of NSCLC patients treated with immunotherapy.

All the previous parameters were calculated by looking at the baseline radiological assessment.

We evaluated those dimensional aspects calculating the following cut-offs with receiver operating characteristic (ROC) curve: maximum lesion diameter (cut-off = 6.3 cm); sum of the 5 major lesions diameters (cut-off = 14.3 cm); number of sites of metastases (cut-off > 4).

We considered as “not measurable” the following lesions: ascites, pleural pouring, pericardial effusion, lung lymphangitis, bone metastases (BM), leptomeningeal metastasis.

We compared survival differences between patients with exclusively thoracic *vs*. extra thoracic disease and with measurable *vs*. not-measurable tumor lesions.

Finally, we compared survival differences between patients with 4 or more sites of metastasis *vs*. those with 3 or less sites of metastasis.

### Statistical analysis

OS was defined as the time interval between the date of first cycle of ICIs administration and death; for patients who had not died, OS was censored at time of last follow-up. PFS2 was defined as the time interval between start of ICIs therapy and tumor progression or death; for patients who had not experienced tumor progression, PFS2 was censored at time of last follow-up. We evaluated PFS2 due to the small number of patients treated with first-line immunotherapy; these patients were excluded from the PFS2 analysis. Survival distribution was estimated using Kaplan-Meier curves and survival differences were evaluated using the log-rank test. Variables that achieved statistical significance (*P* < 0.05) at univariate analysis were included in multivariate analysis using multiple Cox regression to identify independent prognostic factors by calculating their hazard ratios and their 95% confidence intervals (95% CI) [31]. Statistical analysis was conducted using MedCalc software version 19.1 for Windows.

## Results

### Patients’ characteristics

Sixty-five NSCLC patients were retrospectively included in this study.

In the whole cohort, median age at diagnosis was 70 years (range 25–86 years) and 48 (73.8%) of patients were men. Almost all of the patients (93.8%) were current or former smokers. At the start of immunotherapy, 82.6% of patients in our cohort had PS-ECOG lower than 2.

In 56.9% of cases, histologic subtype was represented by non-squamous carcinoma. Forty-four (66.7%) patients showed not-measurable lesions with or without measurable disease and twenty-one patients (32.3%) showed only measurable disease at the time of diagnosis. Thirty-three patients (50.8%) had at least one extrathoracic localization of disease. Ten patients received immunotherapy in first line (Table 1).

**Table 1.** Patients’ clinical characteristics

**Clinical characteristics**		**Patients *n***	**%**
Sex	Male	48	73.8
	Female	17	26.2
Histology	Squamous	28	43.1
	Non-squamous	37	56.9
Age at diagnosis	< 70 years	35	53.8
	≥ 70 years	30	46.2
Smoking	Smokers	16	24.6
	Non-smokers	4	6.2
	Former smokers	45	69.2
PS-ECOG (at start of immunotherapy)	≥ 2	9	13.8
	< 2	56	86.2
Metastases sites	Bone	28	43.1
	Pulmonary	47	72.3
	Adrenal	15	23.1
	Lymph nodes	51	78.5
	Pleural	22	33.8
	Cerebral	8	12.3
	Liver	9	13.8
Disease characteristics	Measurable	21	32.3
	Not measurable	44	67.7
	Extra-thoracic localization of disease	33	50.8
	Thoracic localization of disease	32	49.2
Number of sites of metastases	< 4	48	73.8
	≥ 4	17	26.2
Immunotherapy	First line	10	15.4
	Second line	55	84.6
mOS		23.3 months	
mPFS2		5.3 months	

At a median follow up of 21 months, mOS was 23.3 months while median PFS to immunotherapy in the second line (mPFS2) was 5.3 months.

The median PFS to immunotherapy in the first line was not considered due to the small number of patients.

### Association between clinical characteristics and OS

In the whole cohort of patients, no difference in clinical outcome was observed considering sex, age (considering 70 years as cut-off [14, 32]) and smoking status.

Looking at TB, the cut-off calculated with the ROC curve was 6.3 cm for the largest tumoral lesion’s diameter and 14.3 cm for the sum of the 5 largest lesion’s diameters. No significant difference in OS was noted between subgroups of patients according to TB.

**Figure 1.**
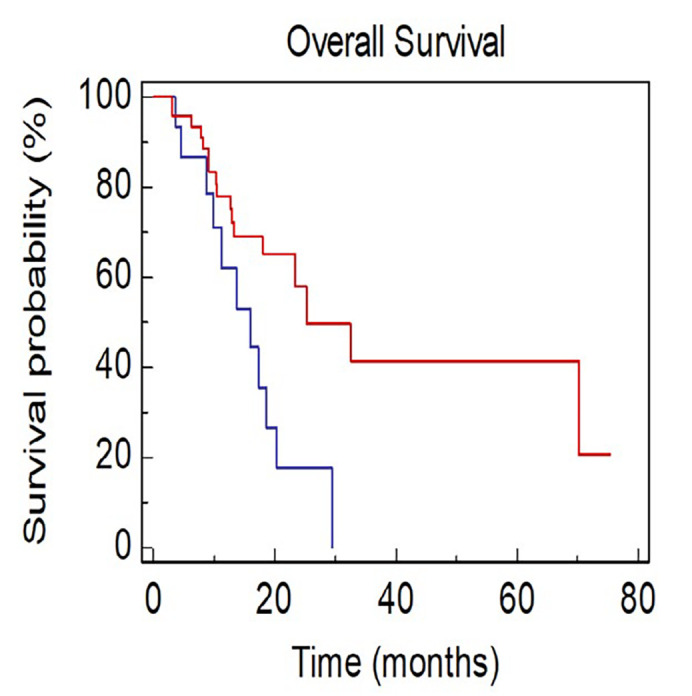
OS of NSCLC patients stratified by number of sites of metastases BLUE ≥ 4 *vs*. RED < 4)

Patients with ≥ 4 metastatic sites showed a worse survival when compared with patients with < 4 metastatic sites [mOS, 15.9 *vs*. 25.2 months; Hazard Ratio (HR): 2.56, 95% CI 1.33–8.63; *P* = 0.0106; Figure 1].

**Figure 2.**
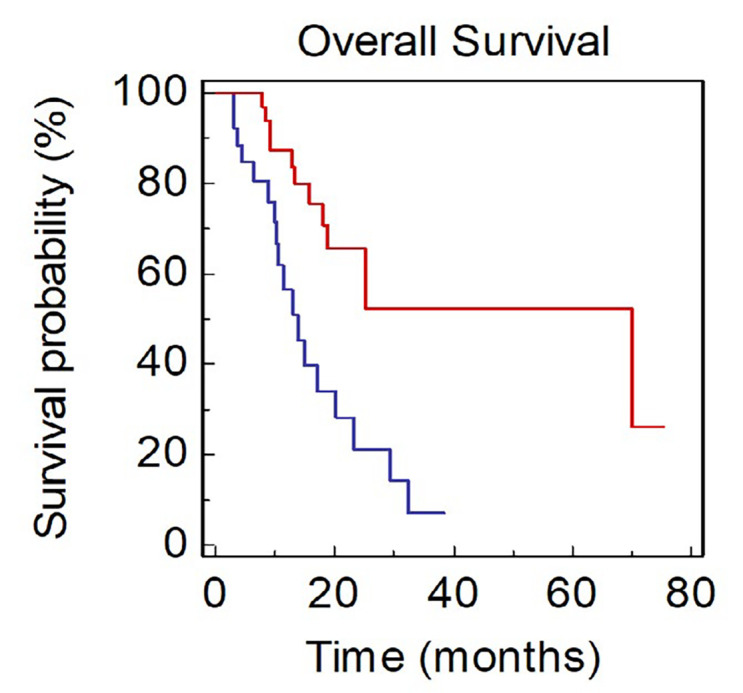
OS of NSCLC patients stratified by PS (BLUE: ECOG ≥ 2 *vs*. RED: ECOG: 0–1)

Patients with a poor PS (PS-ECOG ≥ 2) showed a significantly lower OS compared to those with PS-ECOG 0-1 (mOS, 9.1 *vs*. 24.0 months, HR: 2.43, 95 % CI 1.19–10.56; *P* = 0.0226; Figure 2).

Presence of liver, pulmonary, lymph nodes, pleural, adrenal and cerebral metastases did not result significantly associated with OS.

**Figure 3.**
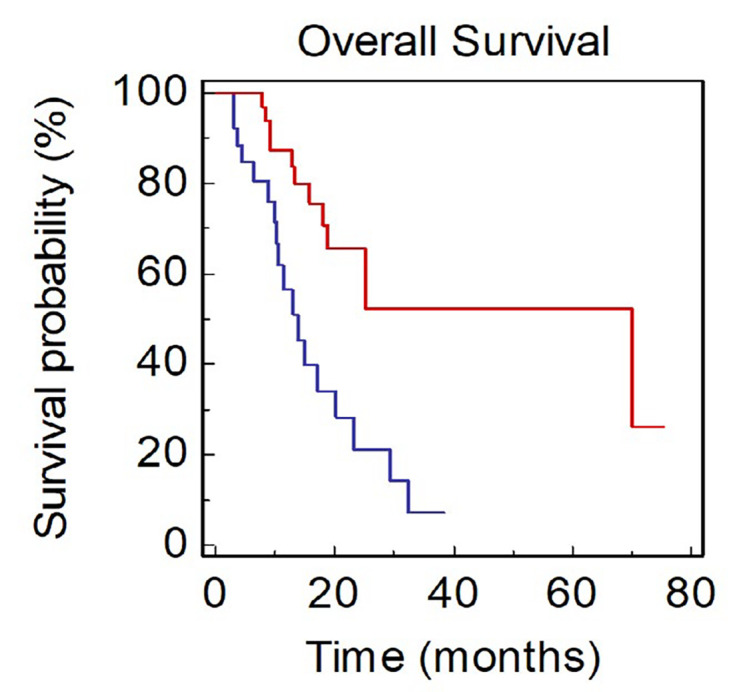
OS of NSCLC patients stratified by BM (BLUE: present *vs*. RED: absent)

Looking at metastatic sites at baseline, presence of BM was associated with a significantly worse OS (mOS, 13.8 *vs*. 70.0 months, HR: 3.27, 95% CI 1.73–8.28; *P* = 0.0009; Figure 3).

**Figure 4.**
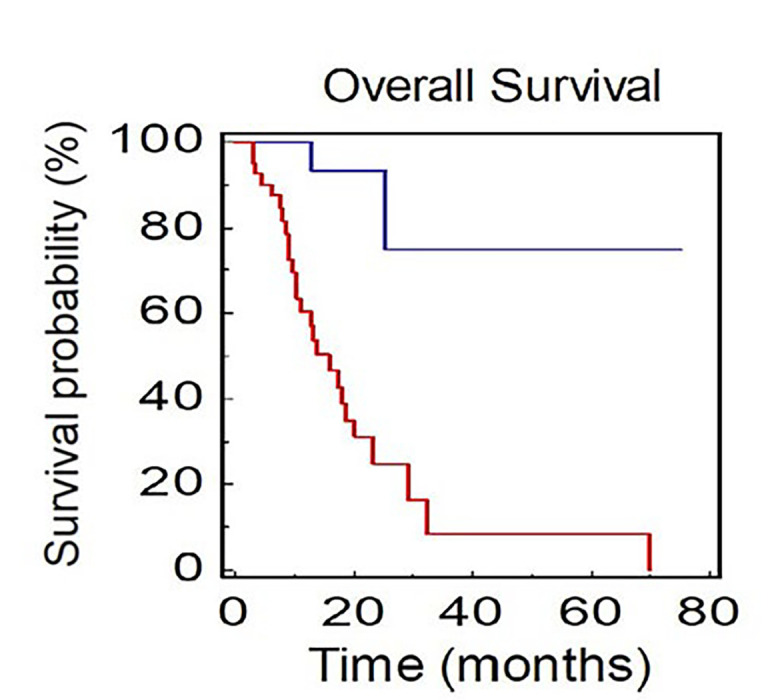
OS of NSCLC patients stratified by presence of measurable or not-measurable disease (BLUE: measurable *vs*. RED: not-measurable)

Patients with not-measurable disease at baseline had a median OS of 15.9 months, while mOS was not reached in patients with only measurable disease (HR: 0.09, 95% CI 0.08–0.44; *P* < 0.0001; Figure 4).

**Figure 5.**
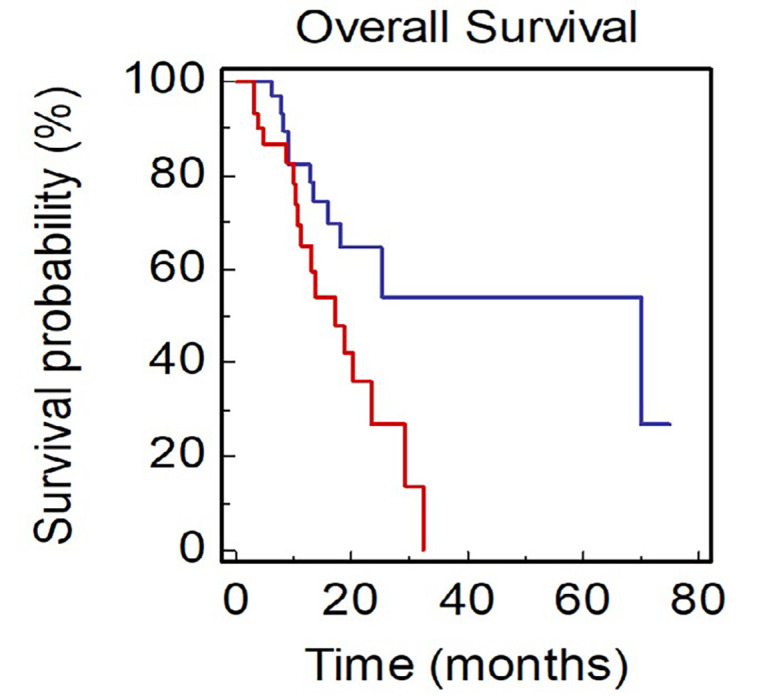
OS of NSCLC patients stratified by site of metastasis (BLUE: thoracic *vs*. RED: extrathoracic)

Patients with only thoracic disease had a better prognosis compared to patients with extrathoracic thoracic disease (mOS, 70.0 *vs*. 17.3 months, HR: 0.40, 95% CI 0.17–0.82; *P* = 0.0136; Figure 5).

The multivariate analysis confirmed BM, non-measurable disease, extrathoracic disease and a number of sites of metastases > 4 as negative independent prognostic factors (*P* < 0.0001) (Table 2).

**Table 2.** Multivariable analyses for OS

**Clinical parameter**	**Univariate cox regression**		**Multivariate cox regression**	
	**HR (95% CI)**	***P*-value**	**Exponentiation of the B coefficient (EXP B, 95% CI)**	***P*-value**
Gender (male *vs.* female )	0.72 (0.30–1.64)	0.4115		
PS-ECOG (≥ 2 *vs.* < 2 )	2,43 (1.19–10.56)	0.0226	0.37 (0.12–1.14)	0.0840
Age (≥ 70 *vs.* < 70 )	1.44 (0.70–2.98)	0.3195		
Histotype (squamous *vs.* non-squamous )	1.08 (0.53–2.21)	0.8328		
Smoke (yes *vs.* no )	0.38 (0.17–3.95)	0.8074		
BM (no *vs.* yes )	3.28 (1.73–8.28)	0.0009	0.19 (0.05–0.78)	0.0222
Pulmonary metastases (no *vs.* yes )	1.08 (0.36–3.23)	0.8845		
Liver metastases (no *vs.* yes )	0.45 (0.11–1.20)	0.0984		
Cerebral metastases (no *vs.* yes )	0.78 (0.20–2.85)	0.6751		
Adrenal metastases (no *vs.* yes )	0.66 (0.22–1.62)	0.3124		
Lymph nodes metastases (no *vs.* yes )	1.29 (0.53–3.33)	0.5502		
Pleural metastases (no *vs.* yes )	0.58 (0.24–1.21)	0.1368		
Maximum lesion diameter (cut-off = 6.3 cm)	0.80 (0.35–1.86)	0.6097		
Sum of the 5 major lesions diameters (cut-off = 14.3 cm)	1.35 (0.46–4.31)	0.5438		
Intrathoracic *vs.* extrathoracic disease	0.40 (0.17–0.82)	0.0136	0.12 (0.03–0.52)	0.0212
Measurable *vs.* not-measurable disease	0.09 (0.08–0.44)	< 0.0001	0.14 (0.02–0.74	0.0216
Number of metastatic sites (≥ 4 *vs.* < 4 )	2.56 (1.33–8.63)	0.0106	0.65 (0.26–0.87)	0.0372

### Association between clinical characteristics and PFS2

Considering the 55 patients receiving immunotherapy as second line treatment, male had a longer PFS2 (mPFS2, 5.83 *vs*. 3.93 months, HR: 0.44, 95% CI 0.13–0.87; *P* = 0.0248).

No difference in clinical outcome was observed considering sex, age (cut-off: 70 years), PS and smoking status.

No significant difference in PFS2 was noted between subgroups of patients according to the largest tumoral lesion’s diameter and for the sum of the 5 largest lesion’s diameters, intrathoracic localization and number of metastatic sites.

**Figure 6.**
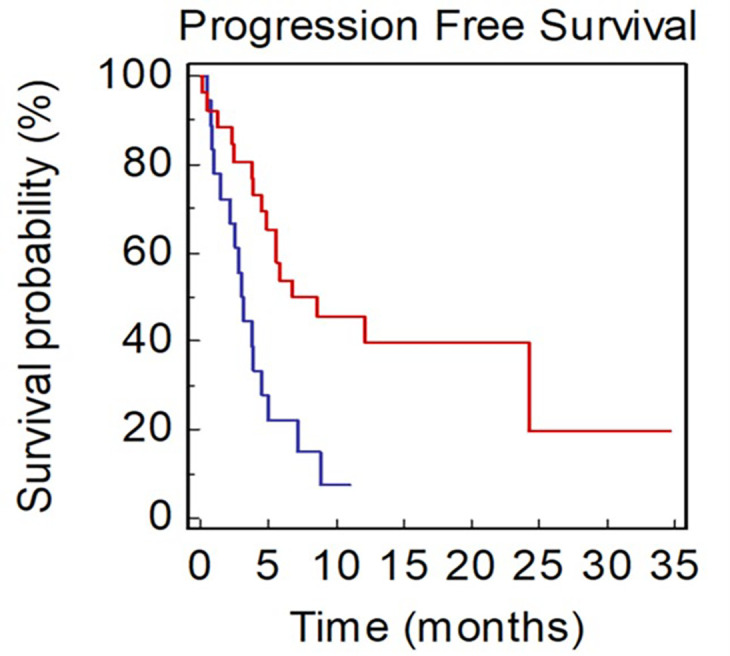
PFS2 of NSCLC patients stratified by BM (BLUE: present *vs*. RED: absent)

Patients with at least one bone metastasis showed a significantly lower PFS2 compared to those without BM (mPFS2, 2.97 *vs*. 8.63 months, HR: 2.60, 95% CI 1.47–7.23; *P* = 0.0037; Figure 6).

Presence of liver, pulmonary, lymph nodes, pleural, adrenal and cerebral metastases did not result significantly associated with PFS2.

**Figure 7.**
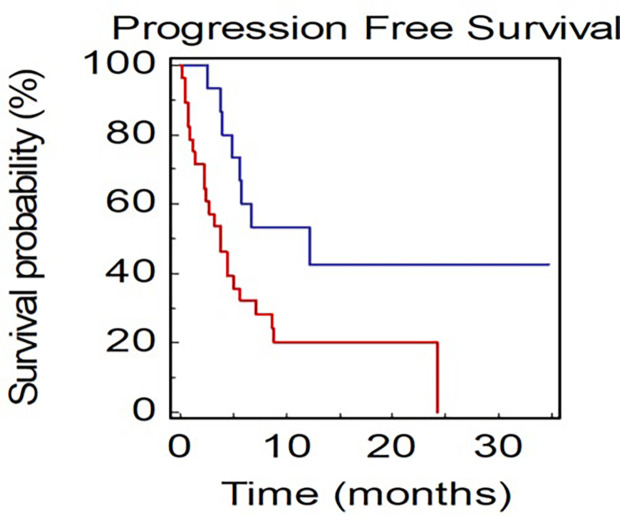
PFS2 of NSCLC patients stratified by presence of measurable or not-measurable disease (BLUE: measurable *vs*. RED: not-measurable)

Patients with not-measurable disease at baseline had a median PFS2 of 3.8 months compared to 12.2 months in patients with only measurable disease (HR: 0.40, 95% CI 0.21–0.87; *P* = 0.0199; Figure 7).

The multivariate analysis confirmed female sex and BM as negative independent prognostic factors (*P* = 0.0016; Table 3).

**Table 3.** Multivariable analyses for PFS2

**Clinical parameter**	**Univariate cox regression**		**Multivariate cox regression**	
	**HR (95% CI)**	***P*-value**	**EXP (B, 95% CI)**	***P*-value**
Gender (male *vs.* female )	0.44 (0.13–0.87)	0.0248	2.56 (1.08–6.07)	0.0340
PS-ECOG (≥ 2 *vs.* < 2 )	1.57 (0. 60–5.06)	0.3039		
Age (≥ 70 *vs.* < 70 )	1.80 (0.87–3.54)	0.1157		
Histotype (squamous *vs.* non-squamous )	1.56 (0.79–3.29)	0.1938		
Smoke (yes *vs.* no )	0.95 (0.22–4.14)	0.9545		
BM (no *vs.* yes )	2.60 (1.47–7.23)	0.0037	0.38 (0.15–0.42)	0.0340
Pulmonary metastases (no *vs.* yes )	1.71 (0.29–1.87)	0.5209		
Liver metastases (no *vs.* yes )	0.55 (0.15–1.41)	0.1756		
Cerebral metastases (no *vs.* yes )	0.50 (0.09–1.55)	0.1769		
Adrenal metastases (no *vs.* yes )	1.52 (0.62–3.40)	0.3830		
Lymph nodes metastases (no *vs.* yes )	1.00 (0.43–2.33)	0.9996		
Pleural metastases (no *vs.* yes )	0.85 (0.41–1.77)	0.6585		
Maximum lesion diameter (cut-off = 6.3 cm)	0.74 (0.34–1.64)	0.4687		
Sum of the 5 major lesions diameters (cut-off = 14.3 cm)	0.66 (0.29–1.66)	0.4071		
Intrathoracic *vs.* extrathoracic disease	0.71 (0.33–1.46)	0.3333		
Measurable *vs.* not-measurable disease	0.40 (0.21–0.87)	0.0199	0.58 (0.22–1.54)	0.2808
Number of metastatic sites (≥ 4 *vs.* < 4 )	0.40 (0.20–0.97)	0.4231		

## Discussion

Identifying prognostic and predictive biomarkers and designing rational combination therapy has become crucial in NSCLC patients treated with ICIs.

The standard approach to check responsiveness to ICI therapy in these patients is to detect the expression levels of PD-L1 in tumor tissues but investigating PD-L1 expression has some limitations. Specifically, around 40–60% of patients will not benefit from immunotherapy [18] and a proportion of patients between 4% and 29% will experience hyperprogression [32].

Research is moving toward the detection of reliable tools for predicting treatment efficacy and some studies have evaluated possible biomarkers both on tumor tissue (such as IDO, dMMR, tumor-infiltrating immune cells, mutational load and natural killer cells) and on peripheral blood (such as neutrophil-to-lymphocyte ratio) [16–19]. Nevertheless, these parameters are not always readily available in clinical practice, thus identifying possible useful and easy-to-access predictors of immunotherapy response remains crucial.

The term TB refers to the total mass of tumor tissue carried by a patient with a malignancy, although a universal definition still does not exist.

Previous clinical evidence has shown that low baseline TB is associated with better patients’ outcome in several cancers. For example, in colorectal cancer patients with resected liver metastases, TB was defined as the number and size of metastases. Patients with high TB (cut-off ≥ 8 metastases and/or 5 cm) have a worse prognosis in terms of disease-free survival (DFS) and OS (HR 1.303; CI: 1.084–1.568; *P* < 0.001) [33]. Furthermore, in renal cancer TB has been previously defined as the sum of the major diameters of the target lesions and has a prognostic role for the response to antiangiogenic drugs such as sunitinib or bevacizumab: specifically, for a 1 cm increase in TB the risk of disease progression rises by 5% [26].

Besides, in a post-hoc analysis of KEYNOTE-001 (involving advanced melanoma patients treated with pembrolizumab), a low TB was associated with a better clinical response and a better OS in the univariate analysis. The multivariate analysis confirmed it as an independent predictor of response to pembrolizumab. In addition, the complete response (CR) rate was higher in patients with lower TB, especially when associated with positive PD-L1 expression (CR rate was 37.6% in patients with low TB, 17.8% with medium TB and 4.7% with high TB) [34].

In our analysis we attempted to “quantify” TB by considering the largest diameter of the largest lesion (cut off: 6.3 cm) and the sum of the largest diameters of the 5 major measurable lesions (cut off: 14.3 cm). Unlike previous studies, stratification of patients in our cohort by these parameters did not reveal significant differences in terms of OS and PFS2. In an analysis from the Kobe Universital Hospital [30] which evaluated 58 NSCLC patients treated with immunotherapy from 2015 to 2018, baseline tumor size (BTS) was defined as the sum of the major diameters of the target lesions and the minor diameters of the metastatic lymph nodes (small BTS: below 101 mm; large BTS: above 101 mm). PFS and OS of patients with a large BTS was significantly shorter than that of patients with a small BTS (mPFS, large *vs*. small: 2.07 *vs*. 6.39 months; *P* = 0.44; mOS, large *vs*. small: 5.85 *vs.* 22.28 months; *P* < 0.01).

Regarding sites of metastases, in our study non-measurable disease and extrathoracic disease were negative independent prognostic factors. Conversely, we found that patients with only measurable disease had a better outcome in terms of PFS2 (mPFS2, not measurable *vs*. measurable: 3.8 months *vs*. not reached; *P* = 0.0199). Other studies comparing these two parameters (not measurable *vs*. measurable and extrathoracic *vs*. thoracic disease) in NSCLC patients treated with ICIs are not available in literature.

Interestingly, we found that only bone localization showed an independent negative prognostic value (mPFS2, bone positive *vs*. bone negative: 2.97 *vs*. 8.63 months; *P* = 0.0037). This result is consistent with the previous pre-clinical and clinical data.

On one hand, preclinical studies have shown how the bone microenvironment reduces the activity of CD4^+^ and CD8^+^ T cells [35]. On the other hand, in a recent retrospective study, Kawachi, et al. [36] observed that skeleton sites were not significantly associated with a shorter PFS (HR: 1.00, 95% CI: 0.65–1.52; *P* = 0.986) in NSCLC patients who underwent first-line pembrolizumab. In another study Tamiya et al. [37] examined metastatic sites of NSCLC patients treated with nivolumab and did not find a statistically significant difference in terms of PFS according to the presence of BM. In addition, they demonstrated a worse response in patients with intrapulmonary (mPFS intrapulmonary positive *vs*. intrapulmonary negative: 2.27 *vs*. 3.52 months; *P* < 0.01) or liver (mPFS liver positive *vs*. liver negative: 3.25 *vs*. 1.15 months; *P* < 0.001) metastases.

Remarkably, in the aforementioned study the presence of 3 or more metastatic sites significantly decreased PFS (mPFS, sites < 3 *vs*. sites ≥ 3: 3.67 *vs*. 1.87 months; *P* = 0.002), while in our analysis only patients with 4 or more metastatic sites were associated with worse OS (mOS, sites < 4 *vs*. sites ≥ 4; 25.2 *vs*. 15.9 months; *P* = 0.0106). Noteworthy, a post-hoc analysis of Check-Mate 057 revealed that patients who progressed to nivolumab within the first 3 months had a high TB defined as more than 5 metastatic sites including liver and bone sites [38].

There are some limitations in our analysis. First, we conducted a retrospective analysis. Second, the small sample size (particularly in first line setting) hampers definitive conclusions. Third, the lack of centralization of radiological imaging might have introduced a detection bias. Finally, our population is homogeneous for some characteristics but not for others. In particular, it is unbalanced by gender (male *vs*. female; 73.2% *vs*. 26.8%, respectively) and by line of treatment (first-line 26.8%, respectively) and by line of treatment (first-line *vs*. second-line; 15.4% *vs*. 84.6%, respectively).

In conclusion, immunotherapy has improved cancer patient outcomes worldwide, but most patients still do not achieve durable disease control. Therefore, the selection of patients more likely to benefit from the treatment is crucial. Our study emphasized the negative prognostic value of a poor PS and of the number of metastases, although it did not show a significant impact of BTS on patient outcome.

The role of specific metastatic sites, the presence of non-measurable disease and extrathoracic diseases represent negative prognostic factors, although our case series was too small to draw conclusions.

Large and prospective studies are needed to clarify the usefulness of TB in evaluating the outcomes of NSCLC patients undergoing immunotherapy.

## Abbreviations

ALK: anaplastic lymphoma kinase
BM: bone metastases
BTS: baseline tumor size
CI: confidence interval
ECOG: Eastern Cooperative Oncology Group
EGFR: epithelial growth factor receptor
HR: hazard ratio
ICIs: immune checkpoint inhibitors
mOS: median overall survival
mPFS: median progression free survival
mPFS2: median progression free survival in second line
NMD: not-measurable disease
NSCLC: non-small cell lung cancer
OS: overall survival
PD-L1: programmed death-ligand 1
PFS2: progression free survival in second line
PS-ECOG: performance status-Eastern Cooperative Oncology Group
ROS-1: ROS proto-oncogene 1
TB: tumor burden

## Supplementary materials

The supplementary materials for this article are available at: https://www.explorationpub.com/uploads/Article/file/100243_sup_1.pdf.

## Declarations

### Copyright

© The Author(s) 2021.
