# Supplementary figures and images for: Tumor burden as possible biomarker of outcome in advanced NSCLC patients treated with immunotherapy: a single center, retrospective, real-world analysis
Source: Explor Target Antitumor Ther. 2021 Jun 28;2(3):227–39. doi: 10.37349/etat.2021.00043 (PMC9400786; doi:10.37349/etat.2021.00043)

Supplementary figure 1: flow chart of study population.

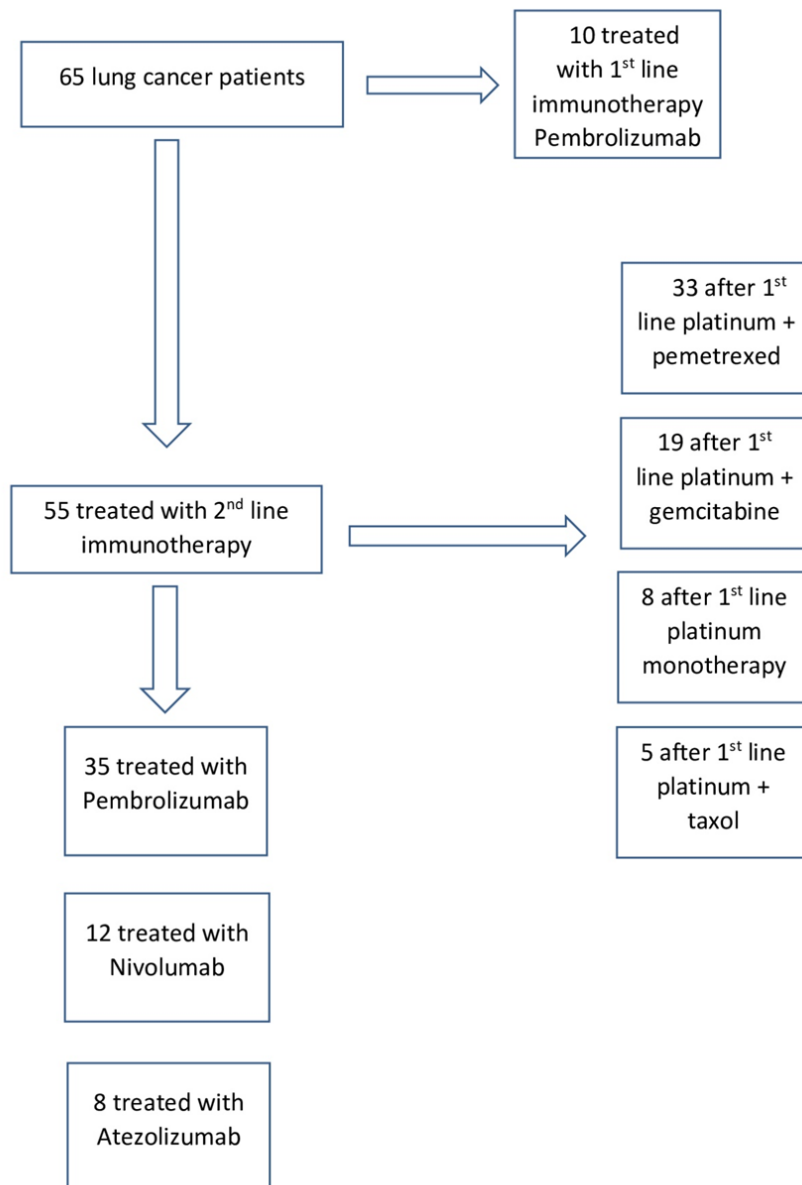

Supplement: Supplementary file 1 [file etat-02-100243-s001.pdf]
